# Supplementary material for: Human attention-guided visual perception is governed by rhythmic oscillations and aperiodic timescales
Source: PLoS Biol. 2025 Jun 27;23(6):e3003232. doi: 10.1371/journal.pbio.3003232 (PMC12204476; doi:10.1371/journal.pbio.3003232)
Supplement: Attachment — Submitted filename: 20250214_Response_to_Reviwers.docx [file pbio.3003232.s011.docx]

Please note the original comments are given in **BOLD** type, while our response is given in *italics*. Changes in the manuscript are highlighted in red.

**Reviewers' Comments:**

**Reviewer #1 (Remarks to the Author):**

**The current work attempts to investigate aperiodic dynamics in behavioral performance and non-invasive physiology (EEG). In a series of simulations and experimentation they claim to be able to dissociate periodic and aperiodic temporal regularities. Following this, they claim that reanalysis of previously acquired data demonstrates that aperiodic temporal structure governs spatial attention.**

**In the context of previous work, this work is reasonably motivated. In what follows I highlight a few concerns that make me less enthused about the interpretation of these results.**

*We thank the reviewer for the positive assessment of our work. We addressed the remaining queries in detail below. However, we respectfully disagree with the assessment that the work is ‘reasonably motivated’.*

*The rhythmic nature of attention has been subject of fierce debate* (Brookshire, 2022; Re et al., 2022; Tosato et al., 2022)*, especially with respect to the question if any underlying aperiodic temporal structure was mistakenly seen as evidence in favor of rhythmic attention models* (Fiebelkorn & Kastner, 2019)*.*

*Here, we contribute to resolving this debate by demonstrating that both, periodic and aperiodic temporal regularities are dissociable and impact different aspects of attention-guided visual perception. Critically, albeit the interest in aperiodic timescales, this is to our knowledge the first report that links neural timescales and behavior, which constitutes the main merit and novel contribution of our work.*

**First, and more pronounced, the authors claim to dissociate two phenomena: periodic and aperiodic temporal structure. This claim is misguided. At a fundamental level, a correlation that is not significant does not provide evidence for the lack of a relationship between the two variables (just like any non-significant statistical inference cannot form, on its own evidence for the null hypothesis). In statistics in order to support the null hypothesis different comparisons have to be devised that enable assessing the likelihood of a relationship between the two variables or the lack thereof. Second, even if we accept the lack of a correlation as evidence for orthogonality – there are several other links that can be demonstrated that are not linear. To make these claims, the authors need to address these options directly using analytic tools or qualify their claims. Presently, in more than one place, the authors provide frequentist statistics and interpret lack of significance as evidence for the null hypothesis.**

**Similar statistical issues come up when different experiments are compared: “we observed that behavioral timescales did not different between both cohorts (Experiment 1a and 2…). This type of statement should be supported by statistical inferences that can provide the likelihood of equivalence.**

*We fully concur with the reviewer and the assessment of the statistical analysis. We therefore complemented all of our statistical claims with Bayes Factor analyses the specifically test for the likelihood of equivalence; hence, supporting the interpretation that the means of the two populations are equal. We incorporated the information into the manuscript in all non-significant instances. All results further support and strengthen the conclusion that periodic and aperiodic temporal regularities do not simply provide redundant information. In addition, we acknowledge the reviewer’s concern regarding potential nonlinear relationships between periodic and aperiodic temporal structures and recognize that alternative forms of dependence could emerge under different methodological approaches. We have now explicitly discussed this point in the limitations section of the Discussion:*

Furthermore, while our results provide no evidence for a systematic nonlinear relationship between periodic and aperiodic temporal structures, future research could further investigate potential dependencies using alternative computational approaches.

**Second, it is unclear why the authors chose to use such a high probability of staying at the current signal value. This results in a signal that is very unlike behavioral or physiological signals, also raising questions over the reported findings.**

*We believe the reviewer is referring to the simulation of solely aperiodic activity. We expanded the methodological description to provide a clearer rationale and motivation. It is critical to highlight that the random walk simulations were designed as a proof-of-concept and constitute an extreme case to illustrate a certain argument and therefore, do not necessarily use biologically plausible parameters. However, we incorporated additional simulations that are more biologically-plausible as outlined below.*

*By using a high probability of staying, we ensured long stability periods, allowing us to test how aperiodic dynamics and timescales emerge under such conditions. Importantly, our conclusions about the dissociation between periodic and aperiodic structures do not rely on the biological plausibility of this specific simulation, but are instead supported by analyses of empirical data. We now state in the Methods:*

First, we simulated pure sine waves in the absence of 1/f noise, in order to isolate the contributions of frequency and amplitude to estimated timescales. Peak frequency was varied from 0.5 Hz to 20 Hz in steps of 0.5 Hz and amplitude was varied across 50 logarithmically spaced values between 0.005 and 0.02 to match the amplitude of the noise signal within plausible limits. Each simulated signal 𝑦(𝑡) was generated as:

$$y\left( t \right)=A\sin(2\pi f\frac{t}{T})$$

Where $t$ represents each time step in the signal, 𝐴 the amplitude, 𝑓 the frequency and 𝑇 is the total signal length.

Second, we generated signals with varying aperiodic 1/f components. These signals were created using a power-law noise model, where the spectral exponent was varied between -4 and -1 across 50 logarithmically spaced values. Each signal 𝑦(𝑡) was generated as:

$$y\left( t \right)=A \eta(t, \beta)$$

where 𝐴 is the amplitude, and 𝜂(𝑡,𝛽) represents a noise signal with a 1/f^β^ power spectrum, where 𝛽 is the exponent. This process produced “colored” noise with a specific amplitude.

Next, we simulated a variety of biologically-plausible time series, which were designed to generate a periodic signal with a specific frequency and amplitude oscillation. We generated a time series that exhibited 1/f characteristics and incorporated an oscillatory component, with exponents ranging from -1 to -4, peak frequency between 1 to 20 Hz and amplitude from 0.005 to 0.02, which scale within biologically-plausible ranges. To simulate the 1/f noise, we created a noise vector with power-law characteristics and we added a sinusoidal oscillation to this 1/f noise to introduce a peak frequency and amplitude component. Each generated signal was defined as:

$$y\left( t \right)= \eta\left( t, \beta\right)+ A\sin(2\pi f\frac{t}{T})$$

*In order to understand whether aperiodic timescales can be detected in the absence of periodic activity, we ran an additional simulation. As highlighted above, it was a strictly hypothetical scenario to convey the idea that timescales can capture temporal regularity even in extreme scenarios where the power spectrum is relatively flat and does not exhibit any distinct oscillatory peaks. We now describe this simulation in more detail in the Methods:*

Lastly, we simulated an aperiodic signal resembling a random walk process with a specific timescale. We employed a Markov process with probabilistic transitions to either maintain the current signal value$(p_{stay})$or introduce a new random value. $p_{stay}$ was set based on the stability period $x$, using the relationship$p_{stay}=1- \frac{1}{x}$. Specifically, each value $y\left( i \right)$ in the signal was generated as:

$$y\left( i \right)=\left\{ \begin{aligned} y(i-1), &with probability 1- \frac{1}{x} \\ randn, &with probability \frac{1}{x} \end{aligned} \right.$$

We used a high probability of staying at the current signal value, as higher values result in more autocorrelation in the generated signal. We generated the random walk signal by iterating through each point $i$ along the signal length and deciding whether to stay at the current signal value or introduce a new random value based on the probability determined beforehand. We then calculated the autocorrelation of the signal as well as the FFT. We repeated this process 1000 times to ensure stability and calculated the mean autocorrelation and spectrum across repetitions.

**Smaller issues:**

**The idea of timescales of aperiodic regularities should and could be better clarified to the reader. And in many places I found the details provided under specified. For example, the “behavioral time scales increase when multiple locations are sampled” figure is missing axes labels.**

*We thank the reviewer for this remark and now provide additional details. We also added the missing axes labels:*

**The following sentence is unclear: “These findings indicate that different analytical parameters have a negligible impact on timescale estimation (<0.03s)” if this means, under 0.03 s there is negligible impact on estimation of time scales this should be spelled out. Otherwise, the parentheses are not clear to the reader.**

*We concur that this statement was unclear, hence, we changed the wording to improve clarity. We now report in the results:*

We re-computed the aperiodic timescales using different window sizes and smoothing windows to examine the effect of averaging window size and smoothing (Figure S1, Methods). These findings indicate that different analytical parameters have a negligible impact of under 0.03s on timescale estimation.

**Since the authors seem to follow contemporary challenges in estimating spectra of time courses (neural and behavioral) might it be relevant to discuss other forms of oscillatory phenomena such as bursts? In averaged data, bursts would appear as 1/f although those are spectral phenomena which are periodic (even if transient).**

*This is an excellent suggestion and we expanded our simulation to separately assess the effect of 1/f noise and oscillatory activity on aperiodic timescales. Reducing the relative contribution of the oscillatory component will render the signal more 1/f-like (even when short bursts are present), hence, reinforcing the idea that timescales can then capture the predominant 1/f dynamics. In addition, we wish to highlight that we have a separate manuscript under review* (Boroujeni et al., 2024) *that relates attention to burst dynamics. Thus, we refrain from further elaborating on burst dynamics in the present manuscript, since this was outside the scope of the current manuscript. We now refer to the burst findings and considerations in the discussion:*

An additional important future direction would be to address other forms of oscillatory phenomena such as high-frequency bursts, which have been shown to support attentional performance and facilitate attentional allocation (Boroujeni et al., 2024).

**Line 389 – missing “period” before the words “Single Neuron…”**

*We changed this accordingly.*

**In the Methods: The parameter for cue to target onset is somewhat unusual for this task and for healthy participants (Exp 1a, 2 and 3). 1000-2000 ms. It would be good practice to indicate what happened on invalid trials. Was the target appearing at the uncued location (I will assume so, but some readers might be mislead to think that perhaps no target appeared on 30 % of the trials). The choice to focus on Reaction Times is a little bit unusual given that the studies cited mostly focus on accuracy**

*We thank the reviewer for this remark. The parameters were chosen in accordance with previously published work. An analysis of the invalid trials was not feasible, since responses were withheld on invalidly cued trials (SNT datasets). In case of the other dataset, we only observed few incorrect trials that did not allow reconstructing timescales estimates. We added this to the limitation section in the Discussion:*

An inherent limitation of the current task design is that participants were instructed to withhold responses to targets at the uncued location, hence, impeding a dedicated behavioral analysis for targets at the uncued location.

**Reviewer #2 (Remarks to the Author):**

**The aperiodic temporal structure of human attention**

**Isabel Raposo et al re-analyzed several behavior and i/EEG datasets which manipulated spatial attention and measured RT at varying cue-target delays. The new analyses focus on the aperiodic structure of behavioral and EEG timecourses. Specifically, the authors computed the decay rate of the autocorrelation function of both i/EEG signals and RT time-courses to capture how quickly the signal autocorrelation drops off, a measure used in single-unit and population recording studies to estimate the timescale of neuronal integration.**

**The main findings are that 1) behavioral timescales increase (RTs correlated with themselves for a longer period into the past and future) when attention is spread across four locations compared to two; 2) scalp EEG timescales during the cue-target interval are associated with RT, such that individuals with shorter timescales have faster RTs; 3) intracranial recordings exhibit small changes in timescale when attention is directed towards an electrode’s preferred spatial location; and 4) that frontal iEEG timescales are shorter when a visual search task is easier (pop-out versus search).**

**Together the paper presents a novel analysis of behavioral and neural timescales using a range of tasks and a range of signal types. However, I find the analysis of timescales, which has a clear interpretation in recordings of single neuron activity, to be a rather vague summary statistic of complex time-varying signals (i.e., behavior changes over time, i/EEG signals containing many component responses) that could change due to many underlying causes. Thus the mechanistic implications for the understanding of attention is somewhat limited. I also have a number of questions and concerns with the data analysis approach and interpretation of results.**

*We thank the reviewer for this encouraging and positive assessment of our work and fully concur that, while the results rely on novel analyses, require additional contextualization, which we provide below.*

**Beginning with the simulation. This is meant to show that when a signal contains periodic (oscillatory) and non-periodic (1/f-like) activity, that the peak frequency and the timescale measure that is derived from that signal are uncorrelated and thus provide distinct information. However, the simulations seem to be based on creating signals where these two features are manipulated independently (i.e., the peak oscillation frequency and the autocorrelation of the aperiodic component) so, unless I am missing something, it’s hardly surprising that they would be uncorrelated given they were simulated as such.**

*We thank the reviewer for this remark. Before we further elaborate on the results, we would like to highlight that we did not manipulate timescales in our modeling. We quantified the timescale while we parametrically modulated oscillatory frequency, amplitude and 1/f noise characteristics. Hence, we did not manipulate both independently.*

**A more convincing simulation would involve a neural network simulation that gives rise to both a periodic and aperiodic component of activity which, may, under more biological constraints, result in a correlation between the two. In other words, the way the signals were generated in the simulation are not likely to reflect the signal generating process in the brain. In general, I also found the details of the simulation to be too sparse in the methods section.**

*We thank the reviewer for this remark. In response, we now provide more details on the simulation in the methods. We also provide additional information as requested by Reviewer #1. A neural network simulation is likewise very parameter sensitive and given that Brookshire* (Brookshire, 2022) *employed simple autoregressive models to challenge the idea of rhythmic sampling, we reasoned that a similar approach is appropriate to refute the incorrect assertions.*

*We fully concur with the reviewer that we a biologically plausible model is more desirable to understand how aperiodic timescales and oscillatory parameters are correlated. We elaborate on the simulation approach.*

*First, we tested the relationship between timescales and oscillatory parameters (frequency and amplitude) in an extreme scenario – without the presence of any (1/f) noise activity. This was achieved by simply simulating sine waves – a scenario that is not biologically plausible. This simulation indicated that timescales do correlate (non-linearly) with oscillatory frequency, but only marginally with oscillatory amplitude.*

New **Figure 1A**. Simulation of a sine wave with different frequency and amplitude peaks. Left: Strong correlation detected between timescales and peak frequency. Right: No correlation observed between timescales and peak amplitude.

*In the Results section we now added:*

To assess whether timescales vary as a function of well-established spectral parameters, such as oscillatory frequency, amplitude or 1/f noise characteristics, we first simulated two extreme scenarios, a purely sinusoidal signal and a purely aperiodic signal. We simulated various oscillations (ranging from 1 to 20 Hz) with varying amplitudes and extracted the timescale from the autocorrelation function. In this physiologically-implausible scenario, we observed that timescales scaled inversely and non-linearly (exponential decay) with the underlying peak frequency (*rho* = -1, *p* < 0.0001; Figure 1A), while oscillatory peak amplitude exhibited no statistically significant correlation (*rho* = 0.05, *p* = 0.7097, BF_10_ = 0.176).

*Second, we repeated this analysis for the relationship between aperiodic 1/f noise and timescales. This scenario is definitely biologically plausible, since several lines of research described that 1/f activity is ubiquitous in the human brain, even when no oscillations are present. Our results show the timescale nicely captured 1/f background noise, irrespective of its amplitude.*

New **Figure 1B**. Simulation of aperiodic 1/f noise with variables exponents and amplitude. Left: Timescales and 1/f exponent were strongly correlated. Right: No correlation observed between 1/f noise amplitude and timescales.

*We now include this in the Results section as follows:*

Secondly, we assessed another extreme scenario, i.e., a signal that exhibited no oscillations, but solely 1/f activity with varying physiologically plausible exponents (ranging from 1 to 4). These simulations indicated that the aperiodic timescales correlated strongly with the 1/f exponent (*rho* = -0.94, *p* < 0.0001; Figure 1B), while the overall amplitude had no significant impact (*rho* = -0.03, *p* = 0.8254, BF_10_ = 0.182). Collectively, these two simulations indicate that timescales correlated with underlying periodic and aperiodic signal features, but did not enable an assessment of relative contributions in signals that mimic empirical timeseries.

*Lastly, we simulated biologically-plausible combinations of 1/f aperiodic and oscillatory activity, which is typically encountered in human electrophysiological recordings. We find that both, 1/f noise and oscillatory amplitude, but not oscillatory frequency, correlate with the neural timescale. Critically, the relationship was much stronger for the relationship between aperiodic activity and the resulting timescale. We do report these results in full now (new* ***Figure 1A-C****) and nuance our language, since timescales are also sensitive (albeit, to a smaller extent) to oscillatory amplitude.*

New **Figure 1C**. Simulation of several timeseries that exhibit both periodic and aperiodic temporal structures with different 1/f exponents, frequencies and amplitudes. Upper left: No statistically significant correlation was observed between timescales and peak frequency of 1000 timeseries with different exponents and oscillations (r = 0.01, p = 0.8305, BF_10_ = 0.045). Upper right: Strong significant correlation was identified between timescales and 1/f noise (r = -0.46, p < 0.0001). Lower left: A moderate correlation between timescales and peak amplitude was observed (r = -0.12, p = 0.0001). Lower right: Bootstrapped and subsample correlation coefficients (100 iterations) reveal a significant difference between the parameters amplitude, frequency and 1/f exponent (F = 406.74, p < 0.0001).

*We have expanded the Results section to include the following:*

Hence, we next simulated timeseries that were more physiologically-plausible and exhibited both, 1/f scaling characteristics as well as sinusoidal oscillations (Figure 1C). From these timeseries, we estimated both periodic neural activity (oscillatory peak above the 1/f decay function) and the aperiodic timescale. We simulated 1000 timeseries with varying physiologically plausible exponents (ranging from 1 to 4) and oscillations (ranging from 1 to 20 Hz). We predicted that both should be correlated if periodic and aperiodic temporal structure provide redundant information. However, we observed no statistically significant association between the peak frequency and the timescale, as assessed using Spearman’s rank correlation (*rho* = 0.01, *p* = 0.8305, BF_10_ = 0.045). However, we observed a moderate correlation between oscillatory peak amplitude and the timescale (*rho* = -0.12, *p* = 0.0001). Critically, we observed a pronounced relationship between 1/f aperiodic activity and the timescale (*rho* = -0.46, *p* < 0.0001). We bootstrapped and subsampled correlation coefficients (100 iterations) to obtain reliable estimates. Using repeated-measures ANOVA, we observed a significant difference between the correlations of the three parameters (*F* = 406.74, *p* < 0.0001). Post-hoc tests reveal that all correlations significantly differ (*p* < 0.0001). To address if timescales and peak amplitude overlapped in their variance explained, we employed a Spearman partial correlation. We observed that the correlation between the timescale and 1/f noise remained unchanged after having accounted for oscillatory amplitude (*rho_partial_* = -0.47, *p* < 0.0001), indicating no significant overlap in variance. In sum, these results demonstrate that the timescale mainly correlates with aperiodic signal features and to a much smaller degree with oscillatory peak amplitude, while no impact of oscillatory peak frequency was observed.

*While these simulations provide an intuitive explanation of the relationship between different spectral parameters and the timescale, they might behave differentially in biological circuits. Therefore, we did complement our analyses with a random walk model that by itself is not physiologically-plausible, but still provides insight into what the timescale captures in addition to spectrally decomposed timeseries. This clarification has been incorporated into the Results section:*

Lastly, we addressed a theoretical question arising from these considerations, namely if a timeseries that contained no periodic activity still exhibits aperiodic temporal regularities. Therefore, we employed a random walk model to simulate a timeseries characterized by a flat power spectrum (without oscillatory peaks), but maintained its autocorrelation structure; hence, exhibited an aperiodic timescale (Figure 1D). This scenario indicates that quantification of aperiodic timescales can capture underlying signal regularities that cannot be obtained from spectrally-decomposed time series data.

*Hence, we believe that neural network models constitute an excellent suggestion for future theoretical work and we now discuss that in the Discussion section and derive testable hypotheses from this consideration:*

Future work involving biologically plausible neural network simulations that generate both, periodic and aperiodic components of activity, reflecting signal generation in the brain more closely, could help provide a deeper understanding about these components are their interaction.

**The main result presented from experiment 1 is that timescales are longer when attending four compared to two locations. The timescale measure is highly derived, though, and it is unclear if this is a stable feature of the behavioral timecourse or simply something occurring at the beginning of the timecourse, as participants are still performing the initial shift of attention. Since no average timecourse is shown across the sample, it’s difficult to tell.**

*We thank the reviewer for this remark and in fact, failed to highlight that this information (regarding various starting points for timescale estimation) was already provided in the Supporting Information. We now highlight the presence of the requested data points in the results:*

We repeated the analyses after shifting the temporal window (50 different random starting points) that was used for estimation of the autocorrelation function to test the stability of aperiodic timescales were stable or whether they were longer at the start of the task (e.g., due to a hazard function) (Figure S1A right).

**It is also somewhat puzzling that there is no correlation between the signal timescale and the power at each frequency (figure 2D) since shouldn’t a signal with longer autocorrelation exhibit more energy at lower frequencies?**

*We fully concur. The observed correlation between the timescale and power at 1 Hz was not significant (rho = 0.02), thus, the experimental data in fact does not confirm the theoretical intuition. One explanation is that signal power reflects the squared amplitude (positive sign), and timescales were calculated on broadband voltage fluctuations. The simulations now provided in Figure 1A-C provide support for this consideration.*

**The methods used to analyze the behavioral timecourse is also unclear. It sounds like the RT timecourse was first derived by sliding a 50 ms window across the RTs and averaging within this window, then a “25-point” boxcar function was used for smoothing, but what does 25-point refer to? Is that a 25 ms window? If so, why is that applied after the 50 ms window?**

*This analysis step was necessary (and in fact, explained in* (Helfrich et al., 2018)*), since patient recordings only allowed to record a limited number of trials, which impeded the reconstruction of an entire time course. After time course reconstruction using a 50ms window, a few missing values remained in the data, which were interpolated using a 25ms boxcar window. Hence, the 25ms was not used to smooth the data per se, but to interpolate missing values as the result of the limited temporal sampling. We now clarify this in the Methods section:*

To interpolate missing data points from the data that resulted from the limited temporal sampling, the traces were further smoothed with a 25-point boxcar moving average.

**This approach also makes it seem like the entire RT timeseries was correlated with itself at different lags to generate the autocorrelation function to estimate the timescale, but if this is the case, I don’t understand the control analysis that “shifted the temporal window (50 different random starting points) that was used for estimation of the autocorrelation function to test the stability of aperiodic timescales”. So was a 50 ms window used to compute the autocorrelation function for behavior? If so, why was a 500 ms used for EEG?**

*The reviewer is correct, but it is critical to highlight that EEG data was in fact sampled at a high temporal resolution, while the behavioral data was reconstructed from individual discrete binary responses by binning. As demonstrated in the supplements, we tested a range of parameters, which had negligible impact on the overall estimates. We also tested if timescales were longer in the beginning (due to e.g., a Hazard function), but found overall consistent estimates irrespective of the starting point. We now better motivate this control analysis in the Results:*

We repeated the analyses after shifting the temporal window (50 different random starting points) that was used for estimation of the autocorrelation function to test the stability of aperiodic timescales were stable or whether they were longer at the start of the task (e.g., due to a hazard function) (Figure S1A right).

**I also wonder if similar dynamics in the timescale are present on invalid trials, this would provide a more robust test of whether manipulating attention is actually impacting the behavioral timescale.**

*As outlined in response to Reviewer #1, an analysis of invalid trials was impeded by the trial design. We discuss this limitation in the Discussion:*

An inherent limitation of the current task design is that participants were instructed to withhold responses to targets at the uncued location, hence, impeding a dedicated behavioral analyses for targets at the uncued location.

**Regarding the EEG data analysis. The conclusion that “timescales are dynamic” is based on the change in timescale measured from the scalp signal after target onset. However, as mentioned, the timescale is a highly summarized measure of the signal and multiple component might be contributing to the measured timescale change. For instance, it is likely that the stimulus onset is causing addition sensory-related activity in the EEG signal that might be at a faster frequency that the ongoing activity. This additional activity might be measured as a decrease in timescale, but it seems just as likely that there was no change in timescale I the underlying neural population, just a new neural population with a lower (sensory) timescale became active because of the stimulus and is mixed in with the signal. This same concern applies to the results from the ECoG dataset, which also reflect a mixture of different neural population activity.**

*We fully concur and would like to highlight that we specifically discuss this limitation in the discussion. As Gao et al.* (Gao et al., 2020) *noticed and more recently discussed in a large review* (Zeraati et al., 2024)*, timescales that are obtained from different imaging modalities do differ, yet, are correlated across methods. To resolve these discrepancies, one would require simultaneous unit and LFP recordings, which were not available in the present datasets. Hence, we note and discuss the above-mentioned papers and highlight the necessary future studies:*

Lastly, it should be highlighted that multiple timescales may simultaneously be present within a given population. Timescales in single unit recordings and EEG/iEEG have been observed across a wide-range of temporal scales between ~2-4 (Ito et al., 2020; Zeraati et al., 2023) and ~50-200 milliseconds (Gao et al., 2020; Murray et al., 2014; Spitmaan et al., 2020; Wasmuht et al., 2018; Zeraati et al., 2023). While the very short timescales might possibly reflect AMPA or GABA receptor time constants, longer timescales may index functional interactions (Lendner et al., 2024). Our current findings focus on the longer timescale amenable for EEG, but similar observations have been made at the level of single neuron recordings as exemplified by the findings by Zeraati et al. (Zeraati et al., 2023), who showed that attention changes timescales in the range from ~100-150 milliseconds but not between ~2-10 milliseconds.

**The correlation between timescale and RT is interesting. I wonder if this is specifically linked to attention though? Is the timescale present before the cue also correlated with RT? Is there a relationship within-subjects that the authors explored such as trials with higher/lower timescales predicting slower/faster RT?**

*This is an interesting question, which could supplement the present results. Since the scope of the current manuscript was on dissociating periodic and aperiodic temporal regularities, we think that the additional analyses would be better suited for a dedicated, separate manuscript in tasks that elicit a broader range of behavior. Given that the ECoG recordings were carried out in patients, we were only able to obtain a relatively small number of trials per condition, which impeded the suggested follow-up analyses.*

**I also worry that the attend in/out analysis of the ECoG may be somewhat circular. Since the electrodes were chosen on the basis of having a high frequency response to the attended location, wouldn’t having higher-frequency activity in a signal lead to lower time-scale on average, given that fast dynamics are both high-frequency and less autocorrelated across time.**

*As outlined in response to the concern that low frequency power and timescales are in fact not correlated, we do not think that this does not constitute a circular analysis, especially, since extraction of HFA activity relied on squaring the amplitude time series (positive sign), while timescales were calculated on raw voltage traces. This is fully in line with the report by Cusinato et al.* (Cusinato et al., 2024) *that broadband and gamma timescales are in fact not correlated and likely, constitute distinct phenomena. We now state in the Discussion:*

Importantly, our results align with previous findings (Cusinato et al., 2024) showing that broadband and gamma timescales are not correlated, suggesting that the observed timescale differences are not simply a byproduct of high-frequency activity but rather reflect complementary neural dynamics.

**The effect size of the attend in/out difference was quite small, and likely only significant because each electrode was treated as independent in the analysis (i.e., DF are inflated). Could the authors bolster this effect by looking at a similar analysis of the EEG data for instance with electrodes that are ipsi/contra to the attended location?**

*We thank the reviewer for this remark. Unfortunately, this analysis proved difficult on scalp EEG data due to the limited spatial resolution, which impedes a reliable mapping of receptive field specific representations (typically done on singe unit and LFP recordings). We share the concern with the reviewer that this is a small effect and acknowledge the need for replication in the discussion. We would like to highlight that Zeraati et al.*(Zeraati et al., 2023) *observed a similar effect in monkey recordings that provided single unit resolution and cite this paper in support of our finding.*

**Finally, I have several concerns about the iEEG pop-out data analysis. The authors hypothesize that a task which requires longer maintenance should lead to brain signals with slower timescales, however the pop-out manipulation is not a very strong or selective manipulation of WM maintenance. There are several other confounding factors that vary between pop-out and search tasks other than WM maintenance such as task difficulty and attentional searching requirements. This makes it very hard to say if the timescale difference is related to maintenance or differential use of attention or decision-making dynamics.**

*We agree with the reviewer that our interpretation is not the only possible scenario, hence, we changed the wording to provide a more balanced and nuanced perspective. We now state in the Discussion:*

Here we observed that timescales are functionally dynamic and change with cognitive demands, i.e., shorten during attentional allocation (Figure 4) and prolong during target maintenance (Figure 5), even though other factors, such as decision-making processes, may have also contributed to these effects.

**MINOR
Figure 2B labels the peak in the red power spectrum as 3 Hz, but it looks lower than the peak of the blue power spectrum which is also labeled 3 Hz.**

*We apologize for the lack of clarification, as we took the peak above the 1/f slope, which in both power spectra corresponds to around 3 Hz. Below we visualize the power spectra after removing the 1/f trend that was indicated in the original figure to illustrate the peak detection.*

**References**

Boroujeni, K. B., Helfrich, R. F., Fiebelkorn, I. C., Bentley, N., Lin, J. J., Knight, R. T., & Kastner, S. (2024). *Attentional Information Routing in The Human Brain* (p. 2024.09.11.612548). bioRxiv. https://doi.org/10.1101/2024.09.11.612548

Brookshire, G. (2022). Putative rhythms in attentional switching can be explained by aperiodic temporal structure. *Nature Human Behaviour*, 1–12. https://doi.org/10.1038/s41562-022-01364-0

Cusinato, R., Seiler, A., Schindler, K., & Tzovara, A. (2024). *Sleep modulates neural timescales and spatiotemporal integration in the human cortex* (p. 2024.09.26.614972). bioRxiv. https://doi.org/10.1101/2024.09.26.614972

Fiebelkorn, I. C., & Kastner, S. (2019). A rhythmic theory of attention. *Trends in Cognitive Sciences*, *23*(2), 87–101. https://doi.org/10.1016/j.tics.2018.11.009

Gao, R., van den Brink, R. L., Pfeffer, T., & Voytek, B. (2020). Neuronal timescales are functionally dynamic and shaped by cortical microarchitecture. *eLife*, *9*, e61277. https://doi.org/10.7554/eLife.61277

Helfrich, R. F., Fiebelkorn, I. C., Szczepanski, S. M., Lin, J. J., Parvizi, J., Knight, R. T., & Kastner, S. (2018). Neural Mechanisms of Sustained Attention Are Rhythmic. *Neuron*, *99*(4), 854-865.e5. https://doi.org/10.1016/j.neuron.2018.07.032

Ito, T., Hearne, L. J., & Cole, M. W. (2020). A cortical hierarchy of localized and distributed processes revealed via dissociation of task activations, connectivity changes, and intrinsic timescales. *NeuroImage*, *221*, 117141. https://doi.org/10.1016/j.neuroimage.2020.117141

Lendner, J. D., Lin, J. J., Larsson, P. G., & Helfrich, R. F. (2024). Multiple Intrinsic Timescales Govern Distinct Brain States in Human Sleep. *Journal of Neuroscience*, *44*(42). https://doi.org/10.1523/JNEUROSCI.0171-24.2024

Murray, J. D., Bernacchia, A., Freedman, D. J., Romo, R., Wallis, J. D., Cai, X., Padoa-Schioppa, C., Pasternak, T., Seo, H., Lee, D., & Wang, X.-J. (2014). A hierarchy of intrinsic timescales across primate cortex. *Nature Neuroscience*, *17*(12), Article 12. https://doi.org/10.1038/nn.3862

Re, D., Tosato, T., Fries, P., & Landau, A. N. (2022). *Perplexity about periodicity repeats perpetually: A response to Brookshire* (p. 2022.09.26.509017). bioRxiv. https://doi.org/10.1101/2022.09.26.509017

Spitmaan, M., Seo, H., Lee, D., & Soltani, A. (2020). Multiple timescales of neural dynamics and integration of task-relevant signals across cortex. *Proceedings of the National Academy of Sciences*, *117*(36), 22522–22531. https://doi.org/10.1073/pnas.2005993117

Tosato, T., Rohenkohl, G., Dowdall, J. R., & Fries, P. (2022). Quantifying rhythmicity in perceptual reports. *NeuroImage*, *262*, 119561. https://doi.org/10.1016/j.neuroimage.2022.119561

Wasmuht, D. F., Spaak, E., Buschman, T. J., Miller, E. K., & Stokes, M. G. (2018). Intrinsic neuronal dynamics predict distinct functional roles during working memory. *Nature Communications*, *9*(1), Article 1. https://doi.org/10.1038/s41467-018-05961-4

Zeraati, R., Levina, A., Macke, J. H., & Gao, R. (2024). *Neural timescales from a computational perspective* (No. arXiv:2409.02684). arXiv. https://doi.org/10.48550/arXiv.2409.02684

Zeraati, R., Shi, Y.-L., Steinmetz, N. A., Gieselmann, M. A., Thiele, A., Moore, T., Levina, A., & Engel, T. A. (2023). Intrinsic timescales in the visual cortex change with selective attention and reflect spatial connectivity. *Nature Communications*, *14*(1), Article 1. https://doi.org/10.1038/s41467-023-37613-7
