# Supplementary material for: Human attention-guided visual perception is governed by rhythmic oscillations and aperiodic timescales
Source: PLoS Biol. 2025 Jun 27;23(6):e3003232. doi: 10.1371/journal.pbio.3003232 (PMC12204476; doi:10.1371/journal.pbio.3003232)
Supplement: Attachment — Submitted filename: 20250519_Response_to_Reviewer.docx [file pbio.3003232.s012.docx]

Please note the original comments are given in **BOLD** type, while our response is given in *italics*. Changes in the manuscript are highlighted in red.

**Reviewers remarks:**

**Reviewer #1 (Remarks to the Author):**

**Overall, Raposo and colleagues provide concise and satisfactory responses to the issues raised by both reviewers. I only have one remaining concern I would consider to be of minor importance:**

**I did not find the authors' response to Reviewer 1's comment regarding the interpretation of absent correlations particularly convincing. The absence of a correlation alone is by no means sufficient to claim distinct processes, and I do think the authors' approach to amend frequency statistics by Bayes factor analyses is a good way to go. However, I was surprised that the authors merely added Bayesian statistics without any further comment - at least a brief interpretation of BF values should be added in case (frequentist) statistical comparisons were found to be non-significant.**

*We thank the reviewer for the positive assessment of our revision. We fully concur with the reviewer that our response to Reviewer 1’s comment could have been better supported. We now consistently interpret the Bayes factor values, whenever frequentist statistical comparisons are non-significant. All instances can be found in following locations: lines 114, 120 page 5; line 132 page 6; line 194 page 7; line 250 page 9; lines 293 and 297 page 10; line 374 page 12. Examples include:*

[…] In this physiologically-implausible scenario, we observed that timescales scaled inversely and non-linearly (exponential decay) with the underlying peak frequency (rho = -1, p < 0.0001; Figure 1A), while oscillatory peak amplitude exhibited no statistically significant correlation with moderate evidence in favor of the null hypothesis (rho = 0.05, p = 0.7097, BF10 = 0.176). […]

[…] These simulations indicated that the aperiodic timescales correlated strongly with the 1/f exponent (rho = -0.94, p < 0.0001; Figure 1B), while the overall amplitude had no significant impact with moderate evidence as suggested by the Bayes factor (rho = -0.03, p = 0.8254, BF10 = 0.182). […]

[…] We replicated the previous behavioral finding (Figure 3A) and found no difference between behavioral timescales of different groups of participants in both the two and four location task, with weak to moderate evidence according to the Bayes factor (Figure 4A top; two locations: t20 = 0.01, p = 0.9934, d = 0.004, BF10 = 0.39; four locations: t19 = 0.39, p = 0.6972, d = 0.18, BF10 = 0.43; two-tailed t-test). […]
